# Bedside Magnetocardiography with a Scalar Sensor Array

**DOI:** 10.3390/s24165402

**Published:** 2024-08-21

**Authors:** Geoffrey Z. Iwata, Christian T. Nguyen, Kevin Tharratt, Maximilian Ruf, Tucker Reinhardt, Jordan Crivelli-Decker, Madelaine S. Z. Liddy, Alison E. Rugar, Frances Lu, Kirstin Aschbacher, Ethan J. Pratt, Kit Yee Au-Yeung, Stefan Bogdanovic

**Affiliations:** SandboxAQ, Palo Alto, CA 94301, USAmax.ruf@sandboxquantum.com (M.R.);

**Keywords:** magnetocardiography, medical devices, quantum sensors

## Abstract

Decades of research have shown that magnetocardiography (MCG) has the potential to improve cardiac care decisions. However, sensor and system limitations have prevented its widespread adoption in clinical practice. We report an MCG system built around an array of scalar, optically pumped magnetometers (OPMs) that effectively rejects ambient magnetic interference without magnetic shielding. We successfully used this system, in conjunction with custom hardware and noise rejection algorithms, to record magneto-cardiograms and functional magnetic field maps from 30 volunteers in a regular downtown office environment. This demonstrates the technical feasibility of deploying our device architecture at the point-of-care, a key step in making MCG usable in real-world settings.

## 1. Introduction

Magnetocardiography (MCG) is a noninvasive imaging technology that has shown promise for improving clinical diagnostics. MCG records the magnetic fields outside the body that result from electrical activity in myocardial fibers [1,2,3]. Magnetic field maps (MFMs) obtained from MCG can provide detailed information about the propagation of electrical activity in the heart with minimal distortion, since magnetic fields propagate unimpeded through tissue.

MCG has already been utilized for diagnosis of ischemic heart conditions, where blood vessels may narrow due to plaque buildup, resulting in an inadequate and non-uniform distribution of blood and oxygen in the heart (i.e., coronary artery disease) [4,5]. Not only can MCG detect subtle changes in magnetic fields due to ischemia, but it can also provide source localization of ischemic regions, aiding with treatment planning [6,7]. Numerous studies have demonstrated that MCG can provide complementary information to electrocardiography (ECG) for more confident identification of ischemic disease using MFMs, which can be parameterized by various features of the magnetic field morphology [8,9,10,11,12]. In certain cases, MCG has been shown to provide high sensitivity in detecting coronary ischemia even when the corresponding ECG results are inconclusive, reinforcing its promise for clinical use [13,14,15]. MCG has also been used to detect non-ST-elevated myocardial infarction (NSTEMI) with higher sensitivity and specificity than a standard 12-lead ECG alone [5,15,16,17]. MCG can also aid with silent ischemia detection in conditions like diabetes, where patients may not present typical symptoms, like acute chest pain [12,18,19]. Further, MCG has been used for noninvasive detection of fetal arrhythmias [20,21].

While these applications for MCG are promising, there are formidable technical challenges in developing a non-magnetically shielded, movable, MCG device that can reject ambient/environmental noise, and provide clinically actionable magnetic field images of disease-state effects. Transient human cardiac signals are 10^5^–10^6^ times smaller than background interference so, to detect heartbeat signals in an unshielded environment, magnetometers must have both large dynamic range and excellent sensitivity. Acquiring clear magnetic field images across the torso further requires magnetic field accuracy in each sensor better than one part in 10^7^, so that tiny differences in magnetic field readings across the chest are properly ascribed to cardiac activity rather than sensor inaccuracy. This accuracy can be achieved through calibration or be intrinsic to the sensor, depending on the type of magnetometer. Table 1 compares candidate sensor types with which MCG recordings have been demonstrated. Clinically actionable magnetic field images of cardiac disease-state effects have only been demonstrated with sensor technologies that incur high monetary and practical costs associated with cryogenics and/or magnetically shielded environments. These challenges have limited the widespread adoption of MCG in bedside settings.

The majority of clinical MCG research is performed using superconducting quantum interference device (SQUID)-based MCG systems, due to their extreme magnetic sensitivity and small sensing volume, enabling excellent magnetic field and spatial resolution, respectively [3,4]. SQUID arrays were used in early demonstrations of MCG imaging for detecting cardiac pathologies. Unfortunately, these high-performance systems incur high costs due to the need for cryogenic cooling and large hospital footprints. Compact, spin-exchange relaxation-free (SERF) optically pumped magnetometers (OPMs) have shown clinical utility for collecting MCG data and can operate without cryogenics [3,14,22,24,25,26]. However, SERF OPMs also incur high costs because they must be operated in large, room-scale magnetic shields to eliminate environmental background fields, including Earth’s magnetic field and ambient power line noise. Inductive coil sensors, which are sensitive to the time-derivative of magnetic fields, have been employed for recordings of MCG, but more studies are required to determine clinical utility [23]. Other types of commercially available magnetometers, including fluxgate and tunneling magnetoresistance (TMR) sensors, are low-cost and can operate without shielding and cryogenics. However, demonstrations of sensitivity required for MCG have only been limited to proof-of-concept experiments [27,28,29].

Scalar OPMs are commercially available magnetometers that do not require magnetic shielding or cryogenics for operation [30,31,32]. The combination of excellent sensitivity, large dynamic range, high accuracy, and technical maturity make them especially promising for integration into a multi-channel, high-resolution MCG device. To date, there have been several demonstrations of unshielded measurements of MCG signals using scalar OPM sensors [31,33,34]. Notably, these demonstrations were performed in quiet environments, far away from sources of magnetic interference and with underlying architectures which lack key features essential for clinical deployment. These include the inability to scale to multi-channel configurations needed for clinical utility, inability to form close sensor arrangement for fine imaging resolution of disease states and, crucially, the inability to reject strong ambient noise sources that overwhelm the cardiac signal in real-world clinical settings.

To this end, we have built a custom, bedside MCG system using an array of scalar OPMs, and successfully recorded magnetic field measurements of the average heartbeat over 300 s, from healthy human volunteers in an ordinary indoor office setting. Ten sensors were arranged in a two-layer array to support gradiometric cancellation of interference and, with signal processing, effectively distinguish true cardiac signals from environmental noise, without magnetic shielding.

Our system successfully overcomes the limitations of previous unshielded magnetocardiography (MCG) demonstrations by employing a multi-channel, closely-packable array capable of strong noise rejection. This presents compelling evidence that a non-cryogenic MCG architecture can approach the bedside in an emergency room or similar noisy medical environment. To reinforce this point, we have deployed this system in a real-world clinic and collected relevant human MCG data in a feasibility study [35], showcasing the capability of our system to realize unshielded, bedside MCG technology in a real, high-noise clinical environment. The present manuscript provides technical details, operation principles, the data processing pipeline, and comparison of measurement conditions of the MCG system which was used in the feasibility study and represents a key milestone in translating MCG from controlled laboratory demonstrations to real-world clinical applications.

## 2. Materials and Methods

### 2.1. Technical Description of the System

A scalar OPM is a quantum sensor that contains a heated alkali vapor, through which a laser beam passes. Due to the quantum properties of the atoms, the intensity of transmitted light is modulated at the Larmor frequency, which is proportional to the environmental magnetic field at the sensor [32]. Thus, the magnetic field can be probed by measuring the frequency of the light modulation. Scalar OPMs are advantageous because they are highly sensitive to weak magnetic fields, have a large dynamic range and can be operated without cryogenics and magnetic shielding. Furthermore, commercially available scalar OPMs are compact, lightweight, and have low power consumption. This makes them ideal for integration into a bedside medical device. Given the orders of magnitude separating the strength of geomagnetic fields (~1 × 10^−6^ T) and cardiac fields (max ~100 × 10^−12^ T), scalar OPMs are sensitive only to those components of the cardiac magnetic field aligned to the total magnetic field vector, which in an unshielded environment is comprised primarily of the Earth’s geomagnetic field. For further details, we refer the reader to Refs. [26,32,36].

**Figure 1:**
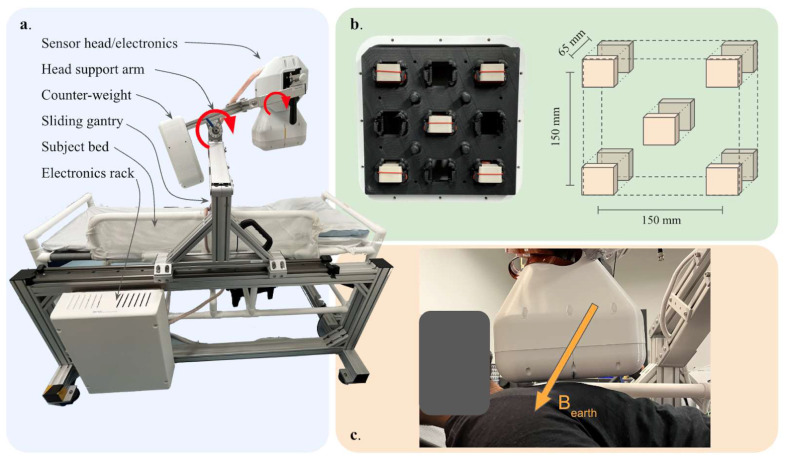
Device overview. (**a**). Photograph of MCG system with critical components indicated. The sensor head and arm can pivot about the points indicated by the circulating red arrows, allowing an operator to position the device optimally over a participant’s chest. Sensors and their control modules are housed within the sensor head assembly, while the data acquisition electronics and other supporting components are placed in the electronics rack indicated at the bottom left. The participant bed is an MRI-compatible hospital-grade bed constructed from non-magnetic PVC. The gantry support is assembled from extruded aluminum. (**b**). (**left**) Photograph of the bottom layer of sensors within the sensor housing. The nonmagnetic, 3-D printed sensor mount can accommodate up to 9 sensors per layer. (**right**) Schematic of both sensor layers indicating dimensions and gradiometric baseline. (**c**). Photograph of a participant with the sensor array positioned for a measurement. The approximate direction of the Earth’s magnetic field is indicated with the arrow labelled B_earth_.

We designed a two-layer scalar OPM array consisting of five pairs of commercial magnetometers (Geometrics MFAM), arranged as shown in Figure 1. To ensure a high common-mode rejection ratio (CMRR), magnetometers were paired as gradiometers with a baseline in the direction normal to the sensor array plane. Since scalar OPMs measure the total magnetic field, the measured field value is independent of sensor orientation (excluding two narrow sensor dead-zones which are avoided through engineering controls). As described in Ref. [30], this enables a high CMRR without the need for strict tolerances on sensor orientation/alignment. The sensor gradiometer baseline (65 mm) was chosen to optimize SNR based on expected cardiac source distances and typical noise source ranges. In this prototype with a limited sensor number, we designed the lateral dimensions of the array to maximize signal coverage, as shown in Figure 1b. Smaller array spacings and larger array extent are expected to increase the performance of denoising algorithms. We applied signal processing techniques (described below) to address the challenge of discriminating between heart signals (typically 10s of pT with a 0.5–40 Hz spectra) and background interference (ranges from pT to uT with broad 0.1–100 Hz spectra).

As shown in Figure 1a, the sensor head is attached to a sliding gantry, which allows it to be adjusted along the length of the participant. The head support arm also includes a double-jointed pivot which allows both coarse and fine rotation of the sensor head. Figure 1b illustrates the spacing between and within sensor planes while Figure 1c shows the sensor head centered above a participant (range 0.5–5 cm) ready for data acquisition.

A 3-lead ECG was recorded simultaneously to provide high signal-to-noise timing triggers to average the MCG data. This custom ECG system was built around a Bluetooth-connected and battery-powered bio-signal acquisition board, paired with non-magnetic surface electrodes (Ambu Neuroline 715).

The prototype can acquire MCG in any overall system orientation, with respect to the cardinal directions. However, given the total field measurement of scalar OPMs, this orientation affects the vector component of the cardiac field that is recorded on the device. MCG in practice is most frequently recorded with the cardiac field component that is normal to the chest plane, which corresponds to this device aligned with the geomagnetic north-south axis.

**Figure 2:**
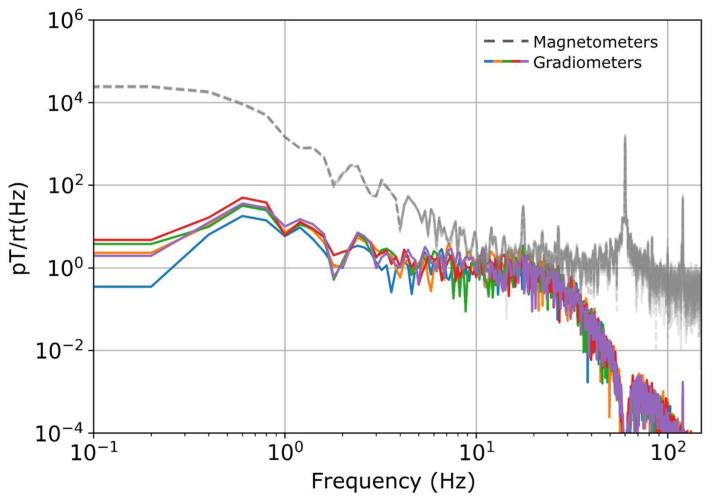
Typical power spectral density (PSD) plot of the unshielded system. Magnetometer signals are shown in grey dashed lines. Filtered gradiometer signals are shown in colors. The PSD is calculated via Welch’s method with a Hann window and normalized by the noise bandwidth. Low frequency environmental noise and 60 Hz line noise dominate the magnetometer signal. These are effectively reduced by bandpass filtering, notch filtering and gradiometry, as described in Section 2.2.

A typical power spectral density plot of the unshielded system is presented in Figure 2. The OPMs have a specified bandwidth of 400 Hz.

### 2.2. Data Acquisition and Signal Processing

Control of the system is managed by a custom Python GUI, which initializes and synchronizes data acquisition from 5 control modules and an ECG controller. The control software also logs session metadata and provides real-time, visual indicators to operators for positioning and troubleshooting. Each dataset was tagged with a unique identifier, cataloged according to its associated metadata and processed using custom Python analysis libraries.

**Figure 3:**
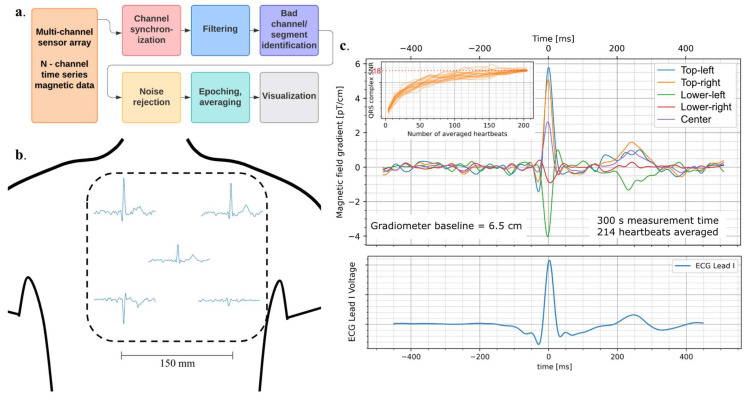
Signal processing pipeline and example data. (**a**). Signal processing pipeline flowchart showing processing steps for time-series data acquired from a multi-channel sensor array. After data are loaded from storage, channel synchronization is performed by aligning common signals that were injected in all channels, including the ECG, which is up-sampled to optimize trigger timing. Filtering follows, which consists of a 60 Hz IRR notch filter and 0.5–45 Hz bandpass using a bi-directional Butterworth digital filter. Then bad channels and segments are identified in and removed from the data using automatic power thresholding and basic data checks. The noise rejection step consists of a combination of gradiometry and Principal Component Analysis (PCA), where signal components that have high noise character are removed. MCG epochs are identified using ECG as a trigger, with automated epoch rejection based on signal power and timing criteria. Finally, epochs are averaged together, and the epoch-average is visualized. (**b**). Epoch-average for each gradiometer channel is displayed based on approximate relative positions over the participant’s chest. The upper right sensor and lower left sensor show inverted features. (**c**). (**Upper**) Epoch-average of all five gradiometric signals overlayed. Inset shows SNR scaling as a function of the number of epochs used to average. Each line corresponds to a different ordering of averaging. SNR > 10 can be achieved with 60 s of averaging and reaches 18 after 214 averages. (**Lower**) Corresponding ECG Lead I trace acquired simultaneously with the MCG data.

Figure 3a summarizes the MCG signal processing pipeline.

*Synchronization*: Initial data processing involves using data acquired during a synchronization pulse to up-sample and time-synchronize the ECG to match magnetometer data. This step ensures that ECG and MCG data are reliably synchronized in the time domain and can be used for epoching and subsequent averaging of the MCG signal.
*Filtering*: MCG data from each recording were notch-filtered at 60 Hz using an IRR filter, and bandpass filtered between 0.5 Hz and 45 Hz using a bi-directional Butterworth digital filter.
*Noise rejection*: Signals from vertically (normal to the chest) adjacent sensors were subtracted to form gradiometric signals with a 6.5 cm baseline. To ensure calibration accuracy of the sensors, we used common-mode fields across sensors to measure possible deviations in balancing weights for gradiometry. To do so, linear-regression was performed on sensor pairs. In practice, the balancing coefficients were all equal to 1 within the expected calibration limits of the sensors, confirming that the sensors were faithfully reporting absolute magnetic field. Principal component analysis (PCA) is a technique which transforms multi-dimensional data into new variables (components) that better capture common variance. In an unshielded environment, common mode variance across sensors is heavily dominated by signals that are common across both the original and gradiometer array. PCA on the original 10 channels can be augmented by making the 5 gradiometry channels available to train PCA filters, while limiting the number of components to 10. In this way, we can capture common mode signals that were imperfectly canceled in gradiometry filtering. Typically, the first two or three principal components are readily identified as noise and removed before reprojecting the components back into signal space. This technique is similar in concept to the signal space projection (SSP) technique [37].
*Epoching*: The ECG data were collected and analyzed in parallel to the MCG in order to identify time segments, or epochs, where heartbeats occurred. ECG lead 1 (LA-RA) was bandpass filtered between 0.5 Hz and 45 Hz using a bi-directional Butterworth digital filter. The filtered ECG was thresholded automatically to find potential QRS times (with false triggers being excluded based on relative timing). These trigger times are further refined by evaluating a 200ms window around the initial guess using a peak-finding algorithm. These triggers were then used to divide the MCG data into epochs of 1000 ms. Epochs were excluded based upon integrated signal power (highest 20% of signal power epochs were excluded) and the remaining epochs were averaged together (Figure 3b,c). For the example data in Figure 3, a total of 214 epochs can be averaged together, but SNR > 10 is possible with as few as 60 epochs (~1 min of data, Figure 3c. inset). The corresponding ECG trace, averaged over 214 epochs, is shown in the lower panel of Figure 3c.

In the currently reported-on system, epoching without the aid of ECG typically recovers fewer than 30% of heartbeats, while using the ECG can identify 100%, or very close to 100% of heartbeats. Unshielded interference is challenging to systematically remove from the magnetometer data without distorting heartbeat morphology. Therefore, after the denoising steps above, the SNR of individual channel time-series data remains insufficient to consistently identify heartbeat timing with the required accuracy for epoch averaging. In ischemia triage, the extra information and reliability makes the tradeoff of adding an ECG subsystem worthwhile.

The resulting epoch-averages on 5 gradiometer channels were fitted using an empirical multi-peak fitting function, designed to capture the QRS and T-wave feature amplitudes, as well as intervals between features. These amplitudes were used to calculate the signal-to-noise ratio (SNR) for each gradiometer channel, using the peak-to-peak amplitude of the QRS complex segment as the signal, and the standard deviation of the segment 200–400 ms prior to the onset of the Q-wave as noise. While this definition leads to reduced SNR reporting than a more standard power-based estimation may give, it better captures the feasibility of feature extraction. We define SNR_max_ as the highest SNR across all 5 gradiometry channels in each dataset.

Each dataset was loaded via an automated pipeline that performed the above steps with algorithmically chosen parameters. Datasets in which the automatic pipeline failed to complete (~30%) due to technical issues or due to excessive noise were analyzed manually, using the same processing steps but with manually selected bad segment identification and parameters for denoising.

After completing data collection for this study, group statistics were performed to understand the distribution of SNR across participants and experimental conditions (described below). Specifically, we report statistics on QRS–SNR because it was found that the T-wave SNR is more dependent on the relative positioning of each magnetic sensor with respect to the heart [38]. Given the low spatial resolution of the current prototype system, this means that T-wave SNR is a less reliable metric of system performance (a given positioning of the sensor array may “miss” the T-wave more easily than QRS). This technicality is resolved by increasing the channel count in future iterations of this device.

### 2.3. Investigation Design and Set-Up

We deployed this investigational MCG system in an office environment in close proximity to operational train tracks, power lines, and roads. To evaluate the system in this magnetically unshielded environment, MCG data were acquired from 30 adult participants (multiple MCG sessions per participant). Of these, for statistical mixed modeling analyses, we retained participants who had data available for each of the four conditions, resulting in a subsample of 23 participants and 92 total observations. Participants provided informed written consent prior to collection of MCG data in each session and data were acquired in compliance with all relevant ethical regulations. Individuals were excluded from participation if they had any history of cardiac disease, pacemakers or metal implants in the torso, were pregnant, or breastfeeding. Demographic information was not tracked or controlled given that this was a non-interventional, feasibility demonstration with no demographic group comparisons.

The majority of participants (n = 23) completed four different MCG sessions, each lasting 300 s (5 min), with varying measurement conditions: (i) For resting state (RS) sessions, participants sat quietly for 5 min prior to the scan and remained fully compliant (no speech or movement) throughout the scan. (ii) For elevated heart rate or stressed state (SS) sessions, participants exercised for about 3 min prior to the scan and then remained at rest throughout the scan. (iii) For non-compliant (NC) sessions, participants sat quietly for at least 5 min prior to the scan and were then asked to talk animatedly with the device operators throughout the scan. (iv) Finally, for magnetic interference (MI) sessions, the bed was modified to have a steel sheet affixed to it, which produced large magnetic field artifacts that were amplified by unintentional participant movements, including those associated with heartbeats. The participant instructions were identical to those for the RS measurement.

Prior to MCG data acquisition, participants were instructed to lie supine on the gantry bed with the backrest adjusted to 10 degrees above horizontal. ECG was collected with three non-magnetic surface electrodes (Ambu Neuroline 715), placed at the right wrist (RA), left wrist (LA) and bottom right section of the thorax (Reference electrode). The MCG sensor head was then positioned above each participant’s thorax using a repeatable protocol. Specifically, the central notches of the patient-facing sensor array lid were aligned with a participant’s left-eye and centered on the line connecting their armpits along the perpendicular axis. After centering the sensor array, an average distance of approximately 4 cm was maintained between the front of the sensor panel and each participant’s chest, ample space for participants to breathe normally during MCG acquisition without physically making contact with the sensor panel.

### 2.4. Statistical Analyses

To evaluate the hypothesis that there would be no significant differences in SNR by experimental condition, we utilized mixed modeling (python statsmodels, version 0.14.1). Utilizing the resting condition as the reference group, we compared each of the three other experimental conditions to the reference group using dummy coding. The dummy-coded variables (0, 1) were entered as a fixed effect at Level 1 of the hierarchical linear model, predicting SNR as the outcome, with participants entered as a random effect at Level 2 of the same model. Because this configuration naturally yields three comparisons with the resting state (as the reference group), we utilized a Bonferonni post hoc correction to adjust the critical alpha to 0.017.

## 3. Results and Discussion

A combination of gradiometry and PCA was used to discriminate between heart signals and environmental noise in order to report epoch-averaged MCG waveforms exhibiting clear QRS and T-wave features for the four different experimental conditions (resting state, elevated heart rate/stressed state, participant non-compliance, and increased magnetic interference).

**Figure 4:**
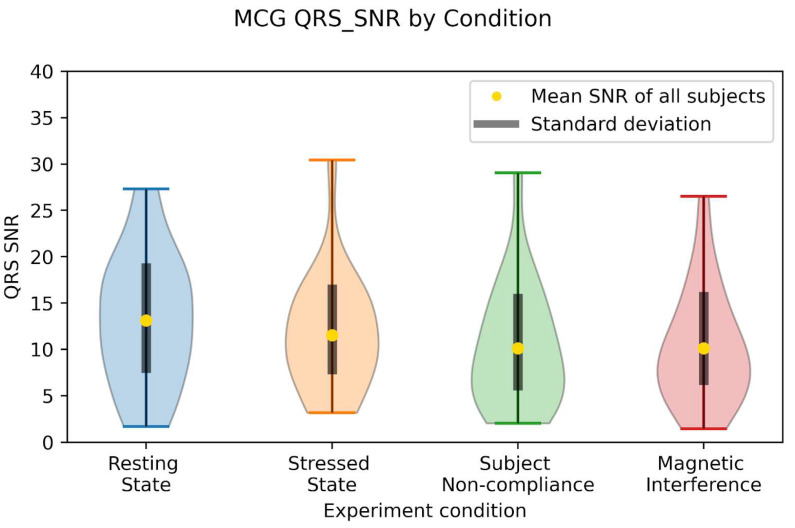
Summary of SNR_max_ of the heartbeat averages, separated by experimental condition for all participants. N_participants = 23, N_observations = 92. Recording length = 300 s. The mean number of heartbeats averaged together for each participant is 191, with a standard deviation of 41. Considering the SNR scaling with number of heartbeats shows that differences in the number of heartbeats averaged cannot account for the spread in SNR values. Wings of each violin plot represent an empirical distribution of the participant results, computed by kernel density estimation (KDE). Mean SNR is indicated for each experimental condition with a gold dot, with the asymmetric standard deviation of the participant level distribution from the mean reported with thick black lines. Mixed modeling comparisons across condition-sorted datasets showed that there were no statistically significant differences in the distributions for each condition, indicating that the controlled factors in the study did not meaningfully affect the SNR.

Of the 104 MCG datasets acquired from the device in this technical feasibility demonstration, 95 (91%) datasets showed clear (SNR > 3) QRS complex among gradiometer channels in the epoch-averaged MCG signal. In the datasets with clear MCG signal visibility and prior to epoch averaging, the typical background white noise rejection ratio was in excess of 100 at 1 Hz, while common mode rejection of narrow band noise overlapping with the MCG signal spectral content was in excess of 1000. The epoch-averaged signal achieved SNR above 20 for 55% of datasets, allowing distinction of QRS and T-wave features that correspond to features in the simultaneously acquired ECG, as illustrated in Figure 3c. In Figure 4, we show the distribution of SNR results for participants across conditions. Mixed modeling analyses revealed no significant differences between any condition and the resting state (all *p* < 0.056 versus a post hoc corrected critical alpha of 0.017). As a result, we accept the hypothesis that the controlled experimental conditions did not significantly influence the SNR distributions.

The mean number of epochs averaged for all datasets was 191 with a standard deviation of 41. On all datasets, we observe a decrease in the background noise that scales as 1/sqrt(N), where N is the number of epochs. This implies that for a fixed signal amplitude, the SNR could vary by up to 11% based on the differences in number of averages. We also observe that SNR reaches 90% of its final value (after all epochs averaged) after 141 epochs, on average. Meanwhile the standard deviation in SNR for the resting state data is nearly 50%. Therefore the variation in number of heartbeats averaged together cannot account for the spread in SNR values. We propose that the variance in SNR across recordings is more likely due to uncontrolled factors, such as variation in operator positioning and participant.

With low spatial resolution, magnetic field maps from which current dipole sources may be accurately estimated are not possible. However, in a qualitative evaluation of the MCG deflection from Figure 3b, we observe inversion of the magnetic field amplitude from the upper right sensor to the lower left sensor. This spatial feature follows from the expected dipolar angle during repolarization for healthy participants [13,17]. The dipolar angle is related to the angle of the equivalent current dipole during the T-wave and has been shown to have sensitivity to ischemic conditions in the heart [17,39].

For this reported-on system operating in challenging environments, data processing without ECG triggering results in SNR lower than 3 in the majority of datasets, while including ECG significantly boosts the SNR. As the device aims to be deployed in clinical settings where the environmental interference will almost certainly be higher, the challenge of extracting single heartbeats will be even greater, even as sensor channels are added and denoising algorithms improve. In applications for our device, such as ischemia triage in emergency departments, the inclusion of a concurrent ECG is not known to impede the clinical workflow while providing a substantial enhancement to the MCG SNR.

## 4. Conclusions

We designed and built a robust, bedside MCG device that could be deployed in real world settings. We achieved this by utilizing an array of scalar OPMs to eliminate the need for magnetic shielding, addressing a key issue that has prevented MCG devices from being widely adopted in clinical practice. After filtering, gradiometry, and PCA, we observed QRS and T-wave features in the heartbeat average time-series waveforms. Demonstration of the device on volunteers over a set of controlled conditions indicated that the SNR of heartbeat-average MCG recordings were not significantly influenced even when participants were not perfectly calm, compliant and without magnetic objects moving in synchronization to their heartbeat. These results indicate that the prototype device design shows promise for future devices that can be robust in real-world conditions, for example, in bedside clinical settings. The validity of the system design is further supported in its deployment to the UCSF hospital cardiology clinic, recording MCG from 25 patients with nurses operating the device [35].

Further increasing the spatial resolution, by increasing the sensor count, will improve the system’s ability to discriminate fine features that may be found in disease-state maps. With higher resolution, magnetic field maps can be generated to identify biomarkers for ischemic disease, and which could allow for improved localization of cardiac current sources. Increased sensor counts would also improve the performance of noise rejection, and future iterations of the system could provide MCG measurements without collecting ECG. Future iterations could also leverage different sensor types or hybrid sensor configurations, given the modular nature of the sensor array and the sensor-agnostic data processing methods we developed.

## Figures and Tables

**Table 1:** Comparison of MCG sensor technologies and readiness for clinical adoption. Point-of-care infrastructure cost is defined as the monetary and practical cost to install and maintain infrastructure associated with a sensing technology. For example, cryogenics or magnetically shielded enclosures incur high capital costs to a hospital or clinic, as well as high logistical costs within a patient’s care pathway if they must transfer outside the emergency department for care. Practical demonstration of clinical utility refers to literature-supported demonstrations of disease classification at or exceeding sensitivity and specificity of the standard of care. The work presented in this publication opens the possibility for deployment of scalar OPMs (underlined last row) to perform such a demonstration of clinical utility.

MCG Sensor Technology	Point-of-Care Infrastructure Cost (Primary Cost Driver)	Practical Demonstration of Clinical Utility
SQUIDs	High (inherent cost + cryogenics)	Yes [4,5,13]
SERF OPM	High (multi-layer magnetic shielding)	Yes [14,21,22]
Inductive coils	Low (system integration)	More Studies Needed ^1^ [23]
Fluxgate	Low (system integration)	No
TMR	Low (system integration)	No
Scalar OPM	Low (sensor)	No

^1^ Clinical utility of QRS complex recorded via magnetic field time-derivative still under investigation.

## Data Availability

The raw data will be made available upon request to the journal. The analysis code needed to reproduce the results from raw data will be made available upon request to the journal.
